# Untangling the Osteopathic Gordian Knot: Reconceptualized Principles for Sustainable and Contemporary Clinical Practice—A Conceptual Perspective

**DOI:** 10.3390/healthcare14091221

**Published:** 2026-05-01

**Authors:** Christian Lunghi, Francesca Baroni, Giandomenico D’Alessandro, Mauro Longobardi, Giacomo Consorti, Nicola Vanacore, Marco Tramontano

**Affiliations:** 1 osteoMANIa: Italian Osteopathic Practice and Research Working Group, BMS Formation, 00146 Rome, Italy; christian@bms-formation.com; 2 BMS Formation, 75116 Paris, France; giandomenico.dalessandro@roi.it; 3 Registry of Osteopaths of Italy, 20149 Milan, Italy; mauro.longobardi@roi.it (M.L.); giacomo.consorti@roi.it (G.C.); 4 Clinical-Based Human Research Department, Foundation Centre for Osteopathic Medicine (COME) Collaboration, 65121 Pescara, Italy; 5 Osteopathy Track and Field Division, Istituto Superiore di Osteopatia, 20126 Milan, Italy; 6 National Center for Disease Prevention and Health Promotion, Italian National Institute of Health, 00161 Rome, Italy; nicola.vanacore@iss.it; 7 Translational Rehabilitation Group, Department of Biomedical and Neuromotor Sciences (DIBINEM), Alma Mater Studiorum University of Bologna, 40138 Bologna, Italy; marco.tramontano@unibo.it; 8 Unit of Occupational Medicine, IRCCS Azienda Ospedaliero-Universitaria di Bologna, 40138 Bologna, Italy

**Keywords:** osteopathic medicine, sustainable healthcare, salutogenesis, person-centered care, interprofessional integration, evidence-informed practice, primary prevention, secondary prevention, tertiary prevention, quaternary prevention, professional identity

## Abstract

**Highlights:**

**What are the main findings?**

- Reconceptualizing osteopathic principles clarifies professional identity and integrates distinctive and interprofessional competencies.
- Osteopathy can be positioned as a person-centered, low-technology, non-pharmacological resource within prevention-oriented and sustainable healthcare frameworks.

**What are the implications of the main findings?**

- Embedding osteopathy within prevention-oriented and territorial healthcare models may reduce over-medicalization and support adaptive capacity.
- Future research and structured consensus-building are needed to guide evidence-informed integration into sustainable healthcare systems.

**Abstract:**

Background: Osteopathy’s integration into contemporary healthcare requires clear articulation of its theoretical and practical foundations and active engagement in interprofessional practice. Despite growing institutional recognition, conceptual ambiguity remains regarding foundational principles and their operationalization. Osteopathy is broadly described as a person-centered, evidence-informed discipline promoting health through manual and educational strategies within systemic and biopsychosocial contexts. Objectives: This Perspective critically examines osteopathic principles, proposes a shared conceptual model for interdisciplinary care, and outlines a structured research agenda for empirical validation, aiming to enhance person-centered, preventive, and sustainable practice. Methods: A narrative review synthesized historical, theoretical, and contemporary evidence. Records were thematically analyzed through expert collaborative brainstorming to achieve consensus, ensuring both conceptual and empirical rigor. Results: Twenty-two studies were included, forming two thematic areas: (1) historical evolution of osteopathic principles, encompassing foundational definitions, early interpretive divergences, codifications, and adaptations; and (2) contemporary reconceptualization for interdisciplinary care, integrating systems-oriented and biopsychosocial frameworks. Emphasis was placed on self-regulation, structure–function relationships, and holistic care. This synthesis bridges historical and modern insights, highlighting osteopathy’s relevance in integrative, pediatric, and preventive healthcare. Conclusions: Reconceptualizing osteopathic principles strengthens professional identity and supports sustainable, evidence-informed, person-centered practice. The proposed framework informs interprofessional collaboration and guides a research roadmap to validate and integrate osteopathy globally within contemporary healthcare systems.

## 1. Introduction

The integration of osteopathy into healthcare systems worldwide requires more than formal recognition; it demands active engagement in interprofessional practice and a clear articulation of the profession’s distinctive theoretical and practical foundations [1]. These elements underpin competency development and role definition and are essential if osteopathy is to contribute meaningfully to contemporary healthcare challenges and service quality [2,3]. Globally, two primary categories of osteopathic practitioners (OPs) are recognized. Osteopathic physicians practice osteopathic medicine, integrating osteopathic manipulative treatment (OMT) into biomedical care, whereas osteopaths provide osteopathic care (OC), typically focusing on OMT and patient education. In the United States, osteopathic physicians are fully licensed medical doctors, trained in both conventional medicine and OMT. In contrast, in many other countries, osteopaths deliver OC without a medical license, generally completing specialized diplomas or degree programs. Internationally, osteopathic education and regulation vary: some countries require statutory registration and ongoing professional development, while others offer voluntary registration with less formal oversight [4]. Nonetheless, despite advances in global regulation, the presence of differing educational pathways, yet all aiming to develop advanced osteopathic competencies, and the increasing institutional integration worldwide, the profession continues to face conceptual ambiguity regarding the interpretation of its foundational principles and the models that should inform clinical practice [2,3].

A degree of convergence has emerged around osteopathy’s broader orientation: it is widely described as a person-centered and evidence-informed discipline engaged in health promotion and management through manual and educational strategies addressing the musculoskeletal system within its systemic context [5]. Clarifying this orientation positions osteopathy within the broader interprofessional landscape, alongside disciplines employing manual or behavioral interventions [3]. However, alignment within a shared healthcare framework does not, in itself, resolve the question of osteopathy’s distinctive professional identity [5]. This unresolved tension has sustained ongoing debate regarding the role of traditional osteopathic principles in contemporary practice. Some authors advocate a tradition-dismissive stance, arguing that historical principles should no longer serve as foundational references [2]. Others contend that these principles remain central to articulating the specific clinical reasoning and therapeutic approach characteristic of OC [2]. A third perspective proposes a reconceptualization of tradition, suggesting that foundational principles should be critically reinterpreted to function as an organizing framework for the profession’s evolving body of knowledge [3]. Against this backdrop, the absence of a shared interpretative framework risks perpetuating fragmentation in professional positioning. A coherent metatheoretical framework is needed to integrate osteopathy’s foundational principles with contemporary scientific and healthcare paradigms [3], while aligning with Sustainable Development Goals (SDGs), such as prevention, health system strengthening, and universal health coverage [6]. Such alignment could help address the shortage of healthcare professionals needed to ensure access to essential and sustainable health services [6]. This framework aims to promote healthy lives and well-being for people of all ages, encompassing key health objectives, including the prevention of non-communicable diseases [6], and protecting individuals from medical interventions that may cause more harm than benefit [7]. This conceptual perspective aims to critically re-examine the application of osteopathic principles, propose a shared model to guide both distinctive and inter-professional practice, enhance the integration of the osteopathic profession within contemporary sustainable healthcare systems, and support the development of a structured research agenda for empirical validation and future refinement.

## 2. Materials and Methods

This study is presented as a conceptual Perspective, a type of article that highlights current developments in osteopathy, emphasizes future directions, and situates the authors’ assessments within the context of relevant literature. It aims to critically re-examine the operationalization of osteopathic principles, propose a shared conceptual model to guide both distinctive and inter-professional practice, and outline a structured research agenda for empirical validation and ongoing refinement. To enhance methodological rigor, elements of narrative review methodology were incorporated in accordance with established guidelines [8]. Unlike systematic reviews, which address narrowly defined questions using predefined criteria, narrative reviews allow integration of diverse sources, including empirical studies, theoretical frameworks, and conceptual reflections, while supporting critical interpretation and contextualization. This approach is particularly suited for a Perspective article, as it enables the exploration of alternative interpretations, the synthesis of historical and contemporary perspectives, and the proposal of novel conceptual directions for osteopathic clinical practice and research. The theoretical framework was developed collaboratively by a team of experts (G.D., C.L., and F.B.), each with over 10,000 h of experience in education, research, and clinical osteopathic practice [9]. It emerged through iterative brainstorming grounded in clinical observations and current evidence, providing a robust foundation for integrating conceptual insights with empirical knowledge.

### 2.1. Research Question

This paper addresses the following research question: How can a re-conceptualization of traditional osteopathic principles, informed by contemporary scientific evidence, clarify and strengthen the role of osteopathic practitioners within inter-professional care systems while contributing to sustainable health and well-being?

### 2.2. Narrative Review Approach

A narrative review approach was adopted to synthesize heterogeneous evidence, historical perspectives, and conceptual insights relevant to osteopathic care [8]. Unlike systematic reviews, which focus on narrowly defined questions and methodologically homogeneous studies, narrative reviews allow broad interpretation, critical analysis, and conceptual synthesis across diverse study designs and theoretical perspectives. This flexibility enables the integration of multiple viewpoints, linking disparate studies to generate a coherent conceptual framework while maintaining methodological transparency and minimizing selection bias. This approach is particularly suitable for Perspective articles, which aim to highlight current developments, emphasize future directions, and reflect the authors’ assessments while situating commentary within the context of recent literature. Narrative reviews acknowledge the interpretive role of the authors, making explicit how clinical, historical, and theoretical expertise shapes the analysis. Insights are therefore grounded in a reflexive and transparent process, helping to mitigate potential bias and ensuring that thematic interpretations are evidence-informed. Based on the aims of this Perspective, the narrative review conducted here can be classified as a critical narrative review with elements of a theoretical integrative review. This subtype enables an interpretative synthesis of heterogeneous literature, combining historical, conceptual, and empirical evidence, while situating the analysis within the authors’ theoretical and clinical expertise. It supports the development of a practical and shareable conceptual framework, bridging foundational osteopathic principles with contemporary interdisciplinary care, and emphasizing both critical appraisal and the advancement of theoretical understanding [8].

### 2.3. Search and Selection

A non-systematic literature search was conducted in PubMed, Scopus, and Google Scholar up to January 2026. To better capture historical literature pertaining to osteopathic principles, additional searches were conducted in electronic databases and digital archives, including Ostmed and the Internet Archive [10]. Given the dual focus on historical foundations and contemporary reconceptualization of osteopathic principles, a comprehensive set of keywords was employed, including: *osteopathic medicine, principles, history, osteopathic philosophy, osteopathic manipulative treatment, Body–Mind Relations, structure–function, palpation, self-regulation, biopsychosocial model, systems medicine, psychoneuroimmunology, free-energy principle, complex systems theory, enactive model, integrative healthcare, personalized medicine, interdisciplinary care, adaptive biological systems, primary prevention, secondary prevention, tertiary prevention, quaternary prevention, culturally sensitive care, evidence-based practice, resilience, adaptation, salutogenesis*. Search terms were adapted to the specific requirements of each database to optimize retrieval [11]. No restrictions were applied regarding study design, population, outcomes, or publication date. Articles were screened in a two-step process (abstract and full-text review) independently by the expert team (G.D., C.L., and F.B.), ensuring consistent application of inclusion criteria. To enhance coverage and mitigate selection bias, additional relevant studies were identified through reference list screening (snowball sampling). The approach explicitly acknowledges that the narrative selection is not exhaustive, but was guided by thematic saturation, conceptual relevance, and the expertise of the research team. Reflexivity was maintained throughout, with continuous discussion among authors to critically evaluate the significance of included studies and ensure that interpretations are transparent, justified, and grounded in evidence.

### 2.4. Eligibility Criteria

The inclusion and exclusion criteria were explicitly justified in relation to the research question, ensuring that each selected record contributed meaningfully to understanding the historical evolution or contemporary reconceptualization of osteopathic principles, and supporting the development of a coherent, evidence-informed conceptual framework. Articles were included if they addressed osteopathic principles, their historical roots and evolution, and their applications in clinical practice, healthcare systems, or preventive care, as well as studies providing contemporary empirical evidence relevant to reconceptualizing osteopathic principles for applied interdisciplinary care. These criteria were explicitly guided by the research question, focusing on how a re-conceptualization of traditional osteopathic principles, informed by contemporary scientific evidence, can clarify and strengthen the role of osteopathic practitioners within inter-professional care systems while contributing to sustainable health and well-being. To capture both historical and contemporary perspectives, the inclusion process considered historical records from specialized archives, alongside contemporary literature retrieved through electronic databases. All study designs were eligible, including empirical studies, commentaries, and relevant grey literature, provided they addressed the phenomenon of interest and contributed to understanding either the historical foundations or contemporary reconceptualization of osteopathic principles. Articles were excluded if they did not align with the core aspects of osteopathic care, namely palpatory and structural diagnosis, holistic principle-based care, and individualized patient-centered approaches. Selection was based on thematic relevance and conceptual sufficiency rather than exhaustive coverage, reflecting the interpretive and iterative nature of narrative reviews. Seminal works and studies with applied relevance were prioritized to ensure a synthesis integrating both historical and contemporary perspectives. All authors critically evaluated each article using a reflective, brainstorming-based model, with iterative cycles of analysis and citation tracking to achieve thematic saturation and conceptual coherence. This process strengthened the development of a robust integrative framework and enhanced transparency and reflexivity in the narrative synthesis.

### 2.5. Synthesis and Critical Interpretation

With reference to the research question, the included studies were organized into thematic domains through critical interpretation within the collaboratively developed framework. A clear distinction was made between articles addressing the historical development and evolution of osteopathic principles, and those providing insights for the reconceptualization and recontextualization of these principles to support osteopathic interdisciplinary care. Limitations and discrepancies across studies were discussed to ensure transparency and academic rigor. To facilitate the systematic development of a practical and shareable conceptual framework, the expert team (all authors) applied a brainstorming-based reflective model, previously used in a recently published Perspective article [2], during structured sessions grounded in the literature findings and clinical experience. This reflective process followed three sequential stages. In the first stage, key themes and insights were identified and summarized from the literature. During the second stage, the significance of these findings was interpreted and their relevance for the development of a practical conceptual framework critically evaluated. Finally, in the third stage, these insights were translated into practical applications and implications for clinical practice, education, and future research. The included papers were then considered for thematic and qualitative analyses, supporting the integrative hypothesis proposed in this Conceptual Perspective. This methodological approach provides a clear bridge to the subsequent Discussion and Conceptual Framework sections, enabling the synthesis of empirical evidence and conceptual reasoning to inform a practical, integrative model of contemporary osteopathic practice.

## 3. Results

A total of 22 articles were included in this narrative review. Two major thematic areas emerged from the synthesis and clinical interpretation of the literature (Table 1): (1) Principles: History and Evolution; (2) Reconceptualizing Osteopathic Principles for Applied Interdisciplinary Care (Table 1).

### 3.1. Principles: History and Evolution

The first thematic area illustrates the historical evolution of osteopathic principles, highlighting foundational definitions, professional codification, and adaptation to medical advances, alongside ongoing interpretive debates (Table 2).

Andrew Taylor Still’s *Our Platform* (1902) [12] articulated nine statements outlining osteopathy’s foundational stance, emphasizing its independence as a system, opposition to drugs, serums, and vaccination, selective use of surgery, reliance on osteopathic measures, respect for sanitation and hygiene, and a commitment to treating disease by correcting anatomical and physiological dysfunctions [12]. These principles established the profession’s core framework and highlighted the early recognition of the need for conceptual clarity. Early graduates such as Barbers interpreted osteopathic principles differently from Andrew Taylor Still and the American School of Osteopathy, emphasizing muscle contractures rather than dislocations and offering alternative educational pathways, including correspondence courses [14]. This period was marked by internal debates on curriculum standards, licensure requirements, and professional legitimacy, as well as tensions with state medical societies. These historical divergences illustrate the long-standing debates on the meaning, teaching, and application of osteopathic principles, highlighting the challenges the profession faced in establishing a unified identity and consistent practice standards. The codification of osteopathic principles began in 1922 with a profession-wide consensus led by Louisa Burns at the A.T. Still Research Institute, which formalized a guiding framework for rational osteopathic treatment [15], including three statements on physiology and health, summarized by a single statement to guide clinicians. In 1953, the Kirksville College of Osteopathy and Surgery further condensed earlier writings into general principles from which are derived a distinctive etiological concept, philosophy, and therapeutic approach [16]. These principles were reaffirmed in 2002 and 2005, emphasizing patient-centered, evidence-informed osteopathic care focused on health maintenance, disease prevention, and holistic integration of body, mind, and spirit [17,18]. The foundational osteopathic principles were codified into four core tenets: (1) the body is a unit; (2) the body possesses self-regulatory mechanisms; (3) structure and function are reciprocally interrelated; and (4) rational therapy is based on an understanding of these relationships [17,18,19,20]. This codification reinforced professional identity, guided the consistent clinical application of OMT, and distinguished osteopathy within modern healthcare [20]. These tenets continue to serve as the central framework for clinical practice, with Gevitz (2006) [20] highlighting that the profession’s long-term vitality depends on embedding them at the core of medical education and patient care, thereby preserving the distinctiveness of osteopathic practices. Although interpretations may vary between traditional and contemporary scientific paradigms, the consistent application of these principles supports a holistic, patient-centered approach and integrates professional training, clinical practice, and identity within the broader healthcare system [19,20]. Although some authors note that osteopathic practitioners have historically expressed reservations about certain interventions, such as immunization, based on the founder’s teachings and traditional perspectives [21], historical records indicate that as early as 1910, during A.T. Still’s presidency, the American School of Osteopathy had already incorporated pharmaceuticals, anesthetics, antiseptics, and vaccines into practice [22]. This demonstrates the profession’s capacity to adapt and integrate osteopathic principles with evolving medical knowledge.

### 3.2. Reconceptualizing Osteopathic Principles for Applied Interdisciplinary Care

The second thematic area reframes these principles considering interdisciplinary and systems-oriented perspectives, showing how core osteopathic concepts support holistic, adaptive care. Together, these findings provide a bridge from historical practice to contemporary applications in pediatric and integrative care (Table 3).

Osteopathic principles and practices are situated at the intersection of anthropological, philosophical, and biomedical traditions, encompassing parallels with indigenous and traditional healing systems [23] as well as contemporary frameworks such as the biopsychosocial model integrated with Ken Wilber’s integral theory and the salutogenesis concept [24]. While the original osteopathic principle refers to “Spirit,” contemporary interpretations apply this concept as “Existence,” emphasizing the integration of body, cognition, and the patient’s personal meaning and sociocultural context to promote self-regulatory capacities. This perspective aligns historical principles with modern clinical practice, fostering a person-centered approach that integrates psychobiological functions with individual values and growth. [23]. Within the profession, tensions persist between ‘traditional-minded’ practitioners, who adhere closely to historical osteopathic principles, and ‘evidence-minded’ practitioners, who align more closely with contemporary manual therapy and biomedical paradigms [23]. Integral and salutogenic approaches extend the biopsychosocial model by integrating subjective and objective, individual and collective, and developmental dimensions of health, highlighting upstream resources and sense-making processes that support well-being [24]. Collectively, these frameworks provide a conceptual basis for osteopathy to deliver culturally sensitive, evidence-informed, and holistic clinical care that bridges historical legacies and contemporary practice. The concept of self-regulation and self-healing, rooted in *vis medicatrix naturae*, underscores the body’s inherent capacity to restore and maintain health, with therapeutic interventions designed to support this process while minimizing unnecessary care [25]. This vitalistic perspective, shared with other integrative health disciplines, promotes patient self-agency, self-efficacy, and reduced dependency on external interventions, reflecting Andrew Still’s assertion that health “comes from within or not at all” [25]. Since the inception of osteopathic medicine, promoting and maintaining health has been central to the profession’s role, and the understanding of health has evolved alongside integration of the biopsychosocial model, psychoneuroendocrinoimmunology (PNEI), and adaptive models of health [26]. OMT combines hands-on approaches targeting somatic dysfunction (SD) with hands-off educational strategies to support homeostatic and allostatic processes, addressing symptoms across biomechanical, neurological, circulatory-respiratory, metabolic, and psychological domains [26]. Structural–functional models guide the interpretation of SD within biological and psychosocial contexts, enabling personalized and adaptive treatments that transcend reductionist, mechanistic approaches [25,26]. The principle of structure–function relationships is deeply rooted in Aristotelian anatomy, which emphasized systematic observation, comparative analysis, and the interdependence of form and function across species [27]. Aristotle’s investigations into morphology, physiology, and behavior illustrated how structural characteristics underpin functional capacities and adaptive responses. Evolutionary theory further situates organisms within ecosystems, demonstrating that form and function emerge through adaptations that optimize energy acquisition and survival in dynamic environments [28]. Computational ecosystem models reinforce this perspective, showing that physical and informational interactions among structural elements produce coherent, emergent behaviors across multiple organizational levels, from proteins to cells to whole organisms [28]. Recent advances in neuroscience integrate these concepts, proposing frameworks such as free-energy minimization, which conceptualizes biological systems as acting to reduce surprise while maintaining adaptability [29]. Within osteopathic practice, structural–functional integration is operationalized as a neuroaesthetic and enactive process, wherein palpatory assessment engages both patient regulative functions and practitioner expertise to co-construct meaning and guide intervention [30]. This approach positions SD evaluation within a holistic, adaptive framework that blends scientific, neurocognitive, and psychosocial understanding with traditional osteopathic tenets, reinforcing the human body as an integrated, adaptive system [27,28,29,30]. OMT addresses movement patterns and SDs, supporting the body’s intrinsic regulatory capacity while promoting systemic adaptation rather than functioning as a purely mechanical intervention [31,32]. The musculoskeletal system serves as a central interface for multisystem integration, shaping perception, agency, and clinical outcomes, consistent with the fourth osteopathic principle of biopsychosocial-existential unity [31,32]. Models such as the Host + Disease = Illness paradigm and the Osteopathic 5 Models illustrate how OMT can modulate host factors and SDs to optimize adaptive responses across neurological, circulatory, respiratory, metabolic, and behavioral domains [31]. Beyond these physical effects, OMT may influence comorbid mental health conditions by generating interoceptive prediction errors that recalibrate central nervous system models of pain and affective states, supporting holistic, person-centered care [32]. In this manner, osteopathic treatment integrates core principles within a complex, adaptive, and multidimensional framework, addressing both somatic and psychosocial health, supporting patient agency, and fostering therapeutic alliance and multidisciplinary collaboration [31,32]. In the following Discussion, the findings from the Results section inform and support the integrative hypothesis proposed in this conceptual perspective, which articulates how osteopathic principles can be applied to meet contemporary healthcare needs and promote sustainable healthcare systems.

## 4. Discussion

This perspective paper identifies two key thematic areas that illuminate the evolution and contemporary relevance of osteopathic principles. The first, “Principles: History and Evolution”, chronicles the historical development, codification, and adaptation of osteopathic principles alongside enduring interpretive debates. The second, “Reconceptualizing Osteopathic Principles for Applied Interdisciplinary Care”, reframes these principles within holistic, systems-oriented, and interprofessional perspectives. Building on these insights, we propose the following Integrative Hypothesis, offering a cohesive framework that bridges historical foundations with modern pediatric, interdisciplinary, and context-sensitive osteopathic practice.

### 4.1. Integrative Hypothesis: Reconceptualizing and Recontextualizing Osteopathic Principles for Contemporary Practice

To operationalize the insights derived from the narrative review, the integrative hypothesis is structured into two interconnected sections that directly reflect the thematic findings of the review, encompassing both the historical evolution of osteopathic principles and their contemporary application in pediatric and interdisciplinary care. Section 4.2 *Historical Development of Osteopathic Principles*, revisits osteopathic principles as dynamic constructs shaped by evolving scientific paradigms and healthcare contexts, and explores their role in defining professional identity and guiding clinical reasoning. Section 4.3 *Reframing Osteopathic Principles for Interdisciplinary Practice*, translates these historical insights into a contemporary framework, detailing how osteopathic principles can inform integrated, person-centered, and evidence-informed care, support systemic and interprofessional practice, and adapt to national and local contexts to promote sustainable, prevention-oriented, and community-based healthcare.

### 4.2. Historical Development of Osteopathic Principles

The need to define and clarify osteopathic principles was recognized early in the profession’s development [12,13]. In 1902, Andrew Taylor Still articulated a foundational platform in Our Platform, outlining nine statements intended to support health and combat disease [12]. From its inception, however, osteopathy confronted the challenge of preserving conceptual coherence while expanding its institutional presence. Divergences in interpretation emerged even among early graduates, including figures such as the Barbers, who distanced themselves from aspects of Still’s philosophy [14]. These tensions illustrate that debate regarding the meaning and application of osteopathic principles is not a contemporary phenomenon, but rather a structural feature of the profession’s evolution. As osteopathy progressively integrated into broader healthcare systems, both in the United States and internationally, it sought to stabilize its identity through formalized articulations of its guiding concepts [12,13,14]. In 1922, consensus efforts codified osteopathic principles as the framework underpinning rational OC [15]. These principles were subsequently condensed in 1953 [16] and reaffirmed in 2002 and 2005, with increasing emphasis on person-centered care [17,18]. Today, they are commonly expressed as four core tenets: (1) the body is a unified entity integrating body, mind, and spirit; (2) the body possesses self-regulatory and self-healing capacities; (3) structure and function are interrelated; and (4) effective treatment is grounded in these concepts [19,20]. Contemporary interpretations of the principles [23] emphasize the integration of body and body representations (i.e., the bridge between inner experiences and the external world), cognition (i.e., the functional system that processes information, coordinates psychobiological processes, and manages rationality), and existence (i.e., the subjective dimension of being, self-awareness, and the non-religious sense of “meaning in life,” including the ways individuals seek and articulate purpose) [3,23]. This approach ultimately promotes adaptability and self-regulatory capacities, supporting the integration of structure and function within social and environmental contexts, and enhancing the manageability of illness (i.e., the patient’s subjective experience), disease (i.e., the objective biological pathology), and sickness (i.e., the social role attributed to the person who is ill) [3,23,26]. Despite their widespread adoption, interpretation and application of these principles remain contested. Some practitioners uphold traditional formulations, whereas others advocate their reinterpretation in light of contemporary scientific paradigms and healthcare demands [2]. Historical debates surrounding medical interventions exemplify this tension. Although Still initially expressed reservations regarding certain practices such as immunization [21], his later acknowledgment of pharmaceuticals, including anesthetics, antiseptics, and vaccines, within the “osteopathic materia medica” reflects an adaptive engagement with evolving medical knowledge [22]. This trajectory suggests that osteopathic principles have historically undergone reinterpretation in response to changing scientific and societal contexts. Viewed through this lens, contemporary discussions on prevention encompass primary, secondary, and tertiary prevention, as well as quaternary prevention and the avoidance of unnecessary interventions [2,3,7,33,34,35,36,37,38], may be understood not as departures from tradition, but as continuations of an ongoing process of conceptual adaptation. Within modern healthcare systems, characterized by diagnostic uncertainty, chronic disease complexity, and risks of overtreatment, osteopathy’s holistic and person-centered orientation may offer a framework for supporting self-management and safeguarding patients from excessive medicalization. Accordingly, ongoing debate should be interpreted not as simple fragmentation, but as a catalyst for reconceptualizing traditional principles. This process safeguards professional identity while ensuring alignment with modern scientific evidence and evolving healthcare demands [2,3,7,33,34,35,36,37,38].

#### Professional Identity and Osteopathic Principles

Professional identity extends beyond technical competence; it encompasses the capacity to embody the core functions, values, and ethical commitments of a profession within specific institutional and societal contexts [39]. In osteopathy, this identity has historically been anchored in its foundational principles and philosophical orientation, particularly in situations characterized by clinical uncertainty and complexity. A recent scoping review on professional identity in osteopathy [40] underscores the centrality of osteopathic principles in shaping practitioners’ self-understanding. At the same time, it identifies structural challenges, such as limited research engagement and insufficient evidence-based practice competencies, that constrain the profession’s integration into mainstream healthcare and may influence the quality and perception of patient care. These findings suggest that identity formation in osteopathy is not only philosophically grounded, but also institutionally mediated. In response to increasing interprofessional integration, some have advocated for a panprofessional practice model grounded primarily in shared healthcare principles. While such an approach may facilitate collaboration, it risks diluting the distinct interpretative framework that has historically characterized osteopathic practice. Educational research indicates that the solution may lie not in choosing between differentiation and integration, but in cultivating a Dual Identity [41]. This model integrates: (1) a profession-specific identity rooted in osteopathic principles and distinctive clinical reasoning, and (2) an interprofessional identity oriented toward teamwork, shared patient-centered goals, and the incorporation of current research evidence. From an educational perspective, this developmental process can be conceptualized through a modified version of Miller’s pyramid [42].

Foundational knowledge (“Knows”) progresses to applied understanding (“Knows How”), where principles-based reasoning reflects competence, and further to observable performance (“Shows How” and “Does”). The apex “Is” captures the internalization of professional values, attitudes, and behaviors, marking the consolidation of identity. In this framework, osteopathic principles function not merely as theoretical statements, but as formative elements shaping professional conduct and clinical judgment. While interprofessional identity has become increasingly well-defined within contemporary healthcare systems, osteopathic professional identity remains characterized by interpretative variability. The absence of a unified, operational framework for translating osteopathic principles into clinical reasoning and practice reinforces this ambiguity [40]. Consequently, the reconceptualization of these principles within a shared interpretative model may represent a necessary step toward strengthening professional distinctiveness while supporting effective collaboration within integrated healthcare environments.

### 4.3. Reframing Osteopathic Principles for Interdisciplinary Practice

When redefining osteopathic principles using modern models, it is important to consider how knowledge is built and used within the osteopathic community. Empirical research suggests that clinicians rarely rely directly on explicit research evidence in isolation when making decisions [43]. Instead, they develop and refine “mindlines”: tacit, collectively reinforced, and contextually negotiated guidelines that shape clinical reasoning. These mindlines emerge through personal experience, dialogue with colleagues, interaction with other professionals, engagement with opinion leaders, and exposure to patients’ narratives, as well as through formal education and research dissemination [43]. If professional knowledge is socially constructed and continuously negotiated, then the reconceptualization of osteopathic principles cannot remain a purely theoretical exercise. Rather, it must be articulated in ways that are communicable, discussable, and adaptable within formal and informal professional networks. Leveraging consensus processes and structured dialogue may help identify misunderstandings, prevent errors, and facilitate culturally sensitive practice grounded in both established principles and contemporary evidence [43]. A second key consideration concerns the interpretation of tradition. In evaluating historical osteopathic thought, whether to preserve, discard, or evolve its tenets, it is important to avoid polarizing narratives that portray early figures either as naïve innovators or as heroic visionaries. Foundational contributors were situated within specific scientific and cultural contexts; they offered valuable insights while inevitably reflecting the limits of their time [44]. Critical engagement with tradition therefore requires historical awareness combined with contemporary scientific literacy. The core osteopathic principles are shared by other medical and philosophical traditions, not just this profession. The principle that the body constitutes a unified entity integrating body, mind, and existence echoes concepts present in indigenous healing traditions [23] and aligns today with salutogenic perspectives, integral views of health, and BPS models of well-being [24]. Similarly, the principle that the body possesses capacities for self-regulation and self-healing can be traced to the concept of *vis medicatrix naturae* [25], and has been reinterpreted through contemporary frameworks such as psychoneuroendocrinoimmunology (PNEI), personalized medicine, and systems medicine [26]. The principle of reciprocal interrelation between structure and function has historical antecedents in Aristotelian comparative anatomy [27] and evolutionary thought, including Darwin’s reflections on proportional organization [28], and has evolved into modern understandings of dynamic form–function interactions within adaptive biological systems [28].

Within contemporary theoretical biology, the Free-Energy Principle (FEP) has been proposed as a unifying account of adaptive, autopoietic, and self-organizing processes, suggesting that living systems act to minimize unpredictability and maintain viable states within their environments [29]. From this perspective, the osteopathic principle of structure–function interdependence may be reconceptualized as a process of predictive optimization within embodied systems [30]. When structural and functional relationships are well integrated, the organism may be better positioned to generate adaptive responses and regulate physiological processes effectively [31]. In this interpretive framework, OC can be understood not as a reductionist mechanical intervention, but as a clinical strategy aimed at supporting adaptive capacity by addressing movement patterns and SDs that constrain systemic regulation. Such an approach situates OC within a broader systems-oriented and predictive understanding of health and disease. The fourth principle, that rational OC, including the biopsychosocial assessment and person-centered interventions, OMT, and educational approach, is grounded in the application of the preceding three principles, thereby acquiring renewed relevance. These principles provide a framework for engaging therapeutically with the individual’s BPS–existential unity, fostering agency through self-regulation and facilitating efficient interaction between structure, function, and environmental context. The centrality of bodily experience in shaping perception and agency is widely recognized [32], and the osteopathic emphasis on the musculoskeletal system in motion reflects an enduring commitment to understanding the body as a primary interface for adaptive interaction [31]. Viewed in this light, the pioneers of osteopathy did not transmit static doctrines, but articulated a distinctive clinical application of broadly shared principles. Their contribution is mainly in how they effectively applied these principles in manual and relational practice, rather than the uniqueness of the principles themselves. To facilitate the transition from historical tenets to contemporary interpretive models, Table 4 presents a synthesized glossary of these reconceptualized principles and their definitions within current conceptual and operational frameworks.

**Figure 1:**
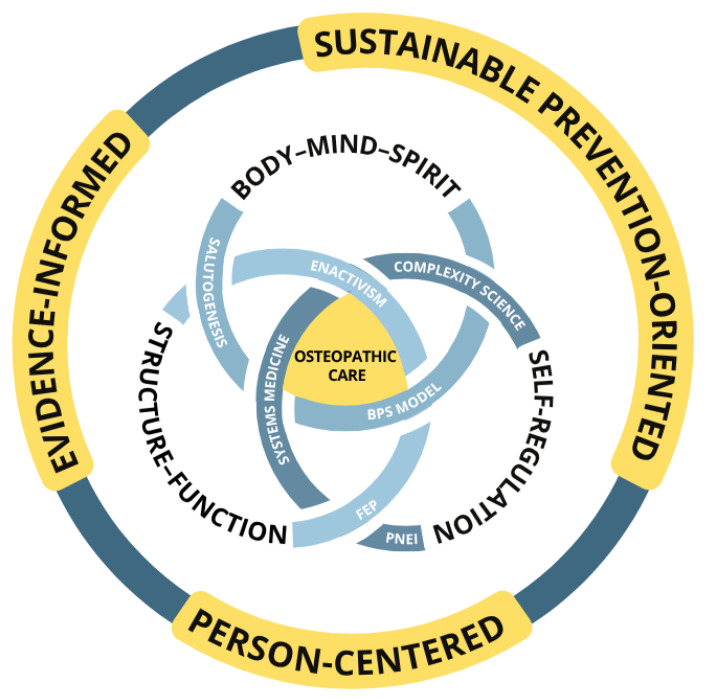
Untangling the Osteopathic Gordian Knot: Reconceptualizing Traditional Principles to Integrate Person-Centered, Evidence-Informed Osteopathic Care into Sustainable Prevention-Oriented Healthcare Systems. BPS: Biopsycosocial model; PNEI: psychoneuroendocrinoimmunology; FEP: Free-Energy Principle.

Contemporary osteopathy can thus contextualize its traditional principles within shared scientific knowledge while maintaining a culturally sensitive and person-centered orientation [23]. The following subsections propose not the abandonment of historical principles in favor of contemporary conceptual models [45], but their integration within current evidence-informed and person-centered frameworks (Figure 1) [2,3], enabling osteopathic practice to remain both distinctive and responsive within modern healthcare systems.

#### 4.3.1. Osteopathic Care in the Light of Reconceptualized Principles

In alignment with the first principle, OC is grounded in the evaluation and treatment of the individual as a dynamic body–mind–existential unity [17,18]. Clinical assessment therefore extends beyond isolated biomechanical findings to include the integration of biological, psychological, and existential domains. Narrative and touch-based sensemaking processes are combined to navigate complex patient presentations and co-construct meaning within the therapeutic encounter [46].

To support this interpretive process in situations of uncertainty and complexity, frameworks derived from complexity science, such as the Cynefin framework, have been proposed to assist practitioners in managing internal contradictions, ambiguous bodily representations, and evolving clinical scenarios, thereby fostering shared understanding between practitioner and patient [2,3]. Within this first-principle orientation, particular attention is given to evaluating individual salutogenesis and adaptive capacity in relation to environmental and contextual factors. Clinimetric criteria provide structured tools for summarizing evaluative and diagnostic standards, rendering complex clinical phenomena assessable through measurable indicators [47]. These criteria help describe and evaluate physical signs, symptoms, and functional patterns that contribute to defining a patient’s health profile. The clinimetric evaluation of adaptive capacity in osteopathic practice, conceptualized as the Osteopathic Allostasis Index, includes social indicators (e.g., significant life events), psychological and existential indicators (e.g., sense of coherence), and biological markers (e.g., anthropometric and metabolic parameters) [46]. These validated criteria are feasible within private practice settings and support clinical decision-making in both initial screening and longitudinal monitoring of health during treatment [47]. In this framework, the integration of salutogenesis allows practitioners to incorporate existential dimensions, understood in non-religious terms as the search for meaning, purpose, and connectedness, alongside biological and social factors, recognizing their role in resilience, identity formation, and adaptive capacity [48]. In accordance with the second principle concerning self-regulation, osteopathic assessment includes evaluation of the patient’s functional profile, considering the potential involvement of multiple regulatory systems in shaping health processes and reported symptoms [47]. During anamnesis, both verbal and embodied narrative elements are collected to identify patterns suggesting dysregulation or adaptive strain. These findings are further examined through functional objective assessments, supported by validated tests addressing biomechanical, neurological, circulatory, respiratory, metabolic, and psychological domains [47]. This integrated evaluation informs clinical reasoning within structure–function osteopathic models. The third principle, emphasizing the interdependence of structure and function, guides focused assessment and management of SD [49]. Manual technical skills enable the identification of tissue adaptations across local and systemic levels, considering asymmetry, movement variability, tissue elasticity, rigidity, and sensitivity [50]. Interpersonal competencies, fostering a therapeutic alliance, support interpretation of the relationship between somatic findings and the patient’s broader functional profile [30]. Within the neuroaesthetic enactive paradigm, structure–function correlation tests contribute to selecting a personalized therapeutic approach through shared decision-making between practitioner and patient, regardless of age [30]. Finally, in accordance with the fourth principle, OMT is grounded in the practical integration of the preceding principles. Treatment strategies are individualized and oriented toward supporting adaptive capacity, promoting self-regulation, and enhancing coherent interactions between structure, function, and contextual demands [3]. In this way, reconceptualized osteopathic principles are operationalized not as abstract doctrines, but as clinically actionable guides for personalized and salutogenic care.

#### 4.3.2. Osteopathic Care and Person-Centered Care

Person-centered care has been framed around three core elements guiding clinical practice: a BPS understanding of the patient’s experience of illness and pain, communication processes that foster shared sensemaking and decision-making, and the promotion of patient engagement and supported self-management [51]. Within this framework, contemporary osteopathy can contextualize its reconceptualized principles by operationalizing them across different phases of patient management [3].

First, consistent with the first and second osteopathic principles, clinicians integrate biological, psychological, social, and existential dimensions through the evaluation of salutogenesis, adaptive capacity, and the individual’s functional profile. This approach situates symptoms within a broader regulatory and contextual landscape rather than isolating them as purely structural dysfunctions. Second, osteopathic practice promotes shared decision-making processes in which bodily representations and experiential narratives serve as central elements for strengthening the therapeutic alliance.

The neuroaesthetic-enactive paradigm (NEP), particularly in the assessment and management of SD, may support this collaborative process by facilitating embodied sensemaking and co-construction of therapeutic goals, in line with the second and third osteopathic principles [30]. Finally, treatment planning reflects the fourth principle through the integration of personalized, salutogenic strategies that combine manipulative and participatory approaches. These interventions are designed not only to address structural and functional imbalances, but also to enhance patient agency, reinforce adaptive capacity, and support long-term self-management [3]. In this way, reconceptualized osteopathic principles align coherently with contemporary person-centered care models, positioning osteopathic practice as both relationally grounded and evidence-informed within modern healthcare systems.

#### 4.3.3. Evidence-Informed Osteopathic Care

Contemporary healthcare increasingly requires clinicians to adopt systematic approaches to clinical problem-solving that integrate the best available research evidence, professional expertise, and patient values [52]. Within this framework, evidence-informed practice represents not an external imposition on osteopathy, but a necessary component of its ongoing professional maturation. With updated osteopathic principles, evidence-informed care integrates research, such as recent neuroscience advances [53], into clinical practice [3]. This process includes synthesizing high-quality evidence regarding outcome measures, reported effectiveness, recommended management strategies, osteopathic techniques described in the literature, session duration and frequency, treatment planning, and follow-up protocols. Rather than applying such data in a rigid manner, the practitioner integrates them within the patient’s clinical context and functional profile, thereby distinguishing empirical evidence from theoretical interpretation. Beyond individual studies, the osteopathic practitioner may also engage with broader healthcare frameworks, including international policy documents [54], national prevention plans [55,56], regional healthcare portals, and local health services that address non-communicable diseases, particularly musculoskeletal conditions and associated systemic symptoms. This alignment situates OC within existing healthcare networks and strengthens its capacity to contribute meaningfully to public health priorities. While this clinically context-centered approach shares common ground with other professions that promote manual therapy [57], it remains anchored in the distinctive interpretative framework articulated through osteopathic principles. Evidence is therefore integrated not as isolated technical guidance, but as part of a structured process that clearly separates empirical data from conceptual application [2,5] and a personalized process that incorporates the practitioner’s expertise and the patient’s embodied experience through shared decision-making [2,3]. The NEP, for example, may support this integration by facilitating collaborative interpretation of bodily representations within clinical reasoning [30]. In this way, the fourth osteopathic principle: “*rational treatment grounded in the preceding principles*” [17,18,20], is operationalized within contemporary socio-healthcare environments while maintaining a clear distinction between empirical evidence and interpretive reasoning. Evidence-informed OC thus represents the convergence of scientific knowledge, clinical expertise, and patient-centered engagement within a coherent and sustainable professional framework.

#### 4.3.4. Reconceptualizing Osteopathy Within Contemporary Healthcare Systems

The transition toward more sustainable healthcare systems increasingly requires a shift from predominantly disease-centered paradigms toward models grounded in salutogenesis, resilience, and person- and community-centered care [58]. Within this evolving landscape, osteopathy occupies a complex position. While its foundational principles appear inherently aligned with person-centered and prevention-oriented approaches, their traditional articulation may at times seem insufficiently connected to contemporary scientific frameworks and to the structural demands of modern healthcare systems [45]. In this sense, “untangling the Gordian knot” of osteopathic identity represents not merely an internal professional exercise, but a strategic step toward coherent integration within global healthcare systems. This discussion proposes that such integration requires a dual process: recontextualization and reconceptualization. Recontextualization acknowledges that osteopathic practice is shaped by national regulatory frameworks, institutional arrangements, and cultural contexts [59]. Reconceptualization, in turn, involves critically updating traditional clinical constructs, such as SD, through contemporary neuroscientific, BPS, and prevention-oriented perspectives [2]. Recent contributions have emphasized the central role of the clinician–patient relationship as a structural enabler of sustainable care [60]. When person-centered care is integrated within broader prevention strategies, it can help limit overdiagnosis, overtreatment, and disease mongering. For example, when applying strategies of quaternary prevention, the focus shifts toward reducing over-medicalization while also addressing systemic constraints such as workforce limitations, environmental impacts, and opportunity costs. In this framework, strengthening meaningful clinician–patient interactions can contribute not only to clinical effectiveness but also to the sustainability of the healthcare system [60]. The following subsections explore these dynamics by examining how national contexts influence the interpretation of osteopathic principles, and how the Italian regulatory framework can serve as an illustrative case of recontextualization within a prevention-oriented and sustainability-focused healthcare model.

#### 4.3.5. The Role of National Contexts in the Reconceptualization of Osteopathic Principles

Osteopathic practice is embedded within diverse national regulatory and professional environments. Codes of practice, scope of competencies, and professional roles vary considerably across countries, reflecting both historical trajectories and specific legal frameworks [59]. Consequently, the interpretation and enactment of osteopathic principles may be shaped, often implicitly, by country-based professional identities. For example, the second principle concerning self-regulation may be operationalized differently in the United States, where osteopathic physicians possess full medical prescribing and surgical authority, compared to non-U.S. contexts where osteopathic practitioners are primarily limited to manual care [61]. Outside the United States, where between 25% and 98% of osteopathic practitioners also hold additional professional degrees (e.g., physiotherapy, human movement sciences, or related disciplines) according to recent surveys [62,63,64,65], prior educational formation may further influence how osteopathic principles are interpreted and translated into clinical reasoning. These variations raise important questions about the degree to which osteopathic identity is shaped by supra-individual regulatory and professional structures rather than solely by shared philosophical tenets. Within the Italian context, for instance, the professional profile emphasizes prevention and positions SD as a central clinical construct, potentially encouraging a more multifaceted and prevention-oriented interpretation of SD compared to other national contexts [30,50,66,67].

Such examples illustrate that osteopathic principles do not operate in a vacuum; they are continuously interpreted within specific socio-legal and institutional environments. In this light, the concept of “recontextualization”, originally used to describe the dynamic relationship between theory and practice [68,69], can be extended to explain how osteopathic principles are operationalized within distinct national settings. Recontextualization involves recognizing how regulatory frameworks, educational systems, economic models, and professional hierarchies influence the practical meaning attributed to foundational principles. Accordingly, advancing a coherent contemporary osteopathic framework requires systematic investigation into how country-specific factors shape the interpretation and application of osteopathic principles; development of collective awareness regarding the normative and organizational positioning of the profession within each healthcare system; and comparative understanding of how osteopathy is framed across different national contexts. In this sense, osteopathic principles may also function as a shared reference point across diverse professional realities. Consistent with the etymological meaning of principium, a beginning or origin, they may serve as conceptual anchors that precede and inform contextual variations. Understanding how these principles are recontextualized in different environments therefore represents a necessary step toward building a structured and internationally dialogical framework capable of supporting further consensus-building and integration within contemporary healthcare systems.

#### 4.3.6. The Italian Context: A Model for Sustainable and Contemporary Practice

The recent regulatory recognition of osteopathy in Italy offers a concrete example of how a prevention-oriented profession can be integrated within a contemporary public healthcare system [70]. According to the Italian professional profile [71], osteopathic practitioners operate both autonomously and collaboratively with other healthcare professionals, focusing on prevention and health maintenance within the musculoskeletal field through the management of somatic dysfunction and the implementation of personalized educational strategies. This legislative positioning aligns with broader global shifts toward resilient and sustainability-oriented healthcare models. However, regulatory recognition alone does not guarantee conceptual clarity, and the professional profile must be interpreted through a contemporary framework that integrates sustainability, prevention, and interprofessional collaboration. Prevention-oriented osteopathic care encompasses all levels of prevention, primary, secondary, tertiary, and quaternary, and is not limited to “healthy” individuals [72]. Grounded in its foundational principles [2], osteopathic care can contribute to quaternary prevention by reducing unnecessary medicalization, hospital length of stay [73,74,75,76,77,78,79], and reliance on pharmacological interventions [77,80], which are particularly relevant in healthcare systems facing economic and organizational strain. In this context, the theory of Adaptive Health Practice complements osteopathic care by providing a personalized approach to health, motivating behaviors that address the behavioral, environmental, and social stressors underlying complex illness, and supporting patients in confronting the challenges associated with maintaining and improving overall health [46]. The Italian context also highlights the need to reconceptualize SD as a dynamic, multifactorial clinical construct rather than an outdated biomechanical notion such as a “misplaced bone.” Recognized within the ICD-11 framework [81], SD operationalizes integrated osteopathic principles [30] and, anchored in contemporary neurophysiological insights and situated within a biopsychosocial and ecological healthcare paradigm [82], can be interpreted as a disruption in adaptive and predictive regulation rather than a static lesion [30]. Osteopathic interventions thus function as a clinical pivot supporting self-regulation and adaptive efficiency.

Importantly, these approaches acknowledge the role of “mindlines,” the tacit, collectively negotiated knowledge guiding practitioners’ clinical reasoning, making implicit processes explicit to strengthen conceptual coherence and guide evidence-informed practice [3]. Although the Italian professional profile emphasizes the musculoskeletal system, this focus should not be interpreted as a limitation. The musculoskeletal system serves as a primary access point through which broader systemic, psychosocial, and existential domains can be engaged [83]. Even the more debated approaches, such as cranial and visceral osteopathy, which act on systems interacting with the musculoskeletal system, can be reconceptualized as body-centered, mindfulness-informed strategies addressing impaired or altered functions of the somatic system, including skeletal, arthrodial, and myofascial structures, along with associated visceral, vascular, lymphatic, and neural elements, to facilitate neural–visceral–sensorimotor co-regulation and patient bio-behavioral synchronization [3]. Within a biopsychosocial framework, manual care, communication, and educational interventions promote functional recovery and psychosocial well-being, thereby supporting individual adaptive capacity [3]. The Italian National Prevention Plan 2020–2025 [56] identifies musculoskeletal disorders and their systemic consequences as a public health priority within broader strategies to address non-communicable diseases, positioning osteopathy within prevention-oriented, territorial, and life-course approaches to care. Emphasizing salutogenesis, patient education, and shared decision-making, osteopathy contributes to bridging individual clinical needs with systemic requirements for sustainable and integrated healthcare. Within this framework, OC also aligns with broader SDGs, including health system strengthening, universal health coverage, and life-course health promotion [84,85]. Overall, the Italian regulatory framework provides not merely a national example, but a model of recontextualization, demonstrating how reconceptualized osteopathic principles can be operationalized within public health priorities, territorial care models, and sustainability-oriented strategies. Although the Italian model for sustainable OC described by various authors is conceptually plausible and aligns with global prevention-oriented frameworks, its practical implementation requires support from an adequate corpus of empirical evidence to substantiate its effectiveness across different levels of prevention.

#### 4.3.7. Evidence Supporting Contemporary Prevention-Oriented Sustainable Osteopathic Care

This subsection highlights selected examples of empirical studies that provide preliminary support for prevention-oriented and sustainable osteopathic care, illustrating how osteopathic interventions may contribute to health promotion and the management of musculoskeletal and systemic conditions. Available evidence suggests potential contributions in maternal health and pregnancy-related musculoskeletal pain [86,87,88], neonatal and pediatric support including preterm infants and feeding-related challenges [73,74,75,76,89,90,91,92,93,94], and work-related musculoskeletal disorders and associated socioeconomic burdens [95,96]. Osteopathic management may also support musculoskeletal health in chronic conditions such as osteoarthritis [97], osteoporosis [98], balance impairment [99], and functional decline in older adults [100]. Primary prevention is operationalized through interventions aimed at maintaining musculoskeletal health and preventing injuries, with OMT shown to improve movement patterns and reduce risk factors such as gait asymmetries and stress fractures in athletes [101,102,103]. Secondary prevention focuses on early management of musculoskeletal issues to prevent progression into chronic conditions, for example, reducing frequency and intensity of tension-type headaches [104]. Tertiary prevention aims to manage established chronic conditions and improve quality of life, demonstrated by reduced hospitalizations and medication use among elderly residents and adults with low back pain [105,106].

Furthermore, the available literature appears to support principle-based OC, grounded in the concept of body unity, as a potential tertiary prevention strategy through whole-body OMT, including cranial and visceral osteopathy. In this regard, a non-controlled before–after clinical study demonstrated positive effects of osteopathic visceral manipulation on quality of life and postural stability in women with endometriosis and women with pelvic organ prolapse [107]. Moreover, a preliminary randomized controlled trial showed that whole-body OMT, focused on body regions associated with SD, including osteopathic visceral manipulations and cranial osteopathy, resulted in improvements in bowel function and quality of life in individuals with spinal cord injuries [108]. Additionally, a systematic review and meta-analysis reported the effectiveness and safety, without major adverse effects, of whole-body OMT, targeting body regions associated with somatic dysfunctions, including visceral and cranial osteopathic techniques, in adults with irritable bowel syndrome [109]. Quaternary prevention targets avoidance of unnecessary interventions, including invasive procedures or excessive medications, where OMT has shown potential to reduce reliance on pain medications and prevent overmedicalization [105,106]. Despite these promising findings, evidence remains limited and inconsistent, particularly in secondary and tertiary prevention [57]. High-quality research is needed to confirm effectiveness and guide clinical practice. Integrating osteopathic care into interprofessional healthcare teams may enhance sustainability by reducing unnecessary healthcare usage and promoting patient empowerment through education, functional neuromyofascial exercises, self-assessment, and collaborative strategies [110,111,112]. This pathway will support the scientific validation and optimal implementation of prevention-oriented, sustainable osteopathic practice, while reinforcing osteopathy’s contribution to global health priorities, including universal health coverage and health system strengthening (SDG 3.8, 3.C, 3.D) [85,113,114,115]. While contemporary conceptual models, including the FEP, enactivism, complexity science, and systems medicine, provide valuable frameworks for understanding osteopathic practice, their empirical validation within osteopathy remains limited, highlighting the need for further research to substantiate these theoretical approaches.

#### 4.3.8. Sustainability, Prevention, and Territorial Healthcare Systems

Contemporary healthcare systems face structural sustainability challenges driven by population aging, increasing prevalence of chronic and multi-morbid conditions, and escalating healthcare demand that cannot be indefinitely absorbed by hospital-centered models of care [116,117]. In response, national and regional health policies are progressively reorienting strategies toward territorial healthcare, prevention, and community-based services, recognizing that acute and highly specialized hospital care, while essential, cannot remain the primary locus of long-term health management [118]. Within this evolving policy landscape, prevention has shifted from a peripheral activity to a structural determinant of healthcare system resilience. Enhancing health literacy, bodily awareness, and self-management capacity is increasingly viewed as a mechanism for reducing inappropriate healthcare utilization, limiting unnecessary diagnostic and therapeutic interventions, and mitigating the long-term costs associated with chronic disease progression [119,120]. From a systems perspective, prevention-oriented approaches contribute not only to improved population health outcomes but also to more appropriate and equitable allocation of finite healthcare resources [121]. The Italian National Prevention Plan 2020–2025 reflects this orientation by embedding prevention within the Essential Levels of Care and emphasizing territorial services, life-course approaches, and citizen engagement as foundational elements of sustainable healthcare [56]. Health is shaped by daily settings, homes, workplaces, and communities, where professionals must work closely with people to spot early vulnerabilities and encourage healthy habits before conditions worsen.

The explicit inclusion of quaternary prevention in contemporary policy frameworks underscores the need to protect individuals from over-medicalization while safeguarding system resources [7,56]. When interpreted through contemporary scientific and public health frameworks, osteopathy aligns with several of these sustainability-oriented objectives. As a low-technology, non-pharmacological, and person-centered practice, osteopathy is structurally suited to territorial settings, where continuity of care, functional monitoring, and patient empowerment are central goals [122]. By focusing on adaptive capacity, movement variability, and BPS-existential dimensions of health, OC may contribute to prevention and quaternary prevention strategies aimed at reducing avoidable healthcare demand and supporting appropriate care pathways. Importantly, this positioning does not imply that OC functions as an alternative to established healthcare professions. Rather, it may operate as a complementary professional resource within interprofessional, prevention-oriented models of care [123]. The sustainability of such models also depends on workforce planning and educational infrastructures. If prevention-aligned professional roles are expected to contribute meaningfully to territorial healthcare systems, their education and training must be understood as public health investments rather than exclusively private responsibilities. This rationale parallels public funding models for other health professions and medical specialization pathways, justified by their strategic relevance to healthcare system resilience [114,124]. From this perspective, supporting university-based education in prevention-oriented professions, including osteopathy, represents a coherent extension of contemporary health policy, ensuring standardized competencies, scientific rigor, and alignment between academic training and the long-term sustainability needs of healthcare systems.

## 5. Limitations

A primary limitation of this work relates to the inherent heterogeneity and discrepancies across included studies. This variability partially stems from the dual focus of our research question, which aimed to explore how the reconceptualization of traditional osteopathic principles, informed by contemporary scientific evidence, can clarify and strengthen the role of osteopathic practitioners within inter-professional care systems while contributing to sustainable health and well-being. Accordingly, our bibliographic strategy was twofold: one search targeted historical and foundational sources documenting osteopathic principles and their evolution over time, conducted largely through digital archives such as Ostmed and the Internet Archive alongside scientific databases (PubMed, Scopus); a second search focused exclusively on contemporary scientific evidence from electronic databases (PubMed, Scopus) to capture the most recent insights relevant to interdisciplinary care and sustainability. This dual approach, while comprehensive, inevitably introduced variability in the types, contexts, and methodological rigor of the included sources. The authors of the present conceptual perspective also acknowledge that the emerging nature of the research topic has led to a higher degree of self-citation, as some of the included studies were authored by the current research team or closely associated collaborators. Nevertheless, these self-citations were selected in accordance with the conventions for Perspective articles, which aim to showcase current developments in a specific field, emphasize future directions, and provide the personal assessment of the authors. While common in nascent fields, this may affect the perceived novelty of the findings and is compounded by the scarcity of recent publications focusing on the unique contributions of osteopathic care. Contemporary discourse has often emphasized perceived limitations of osteopathic care [125,126,127,128] rather than its distinctive strengths and developmental opportunities. Consequently, although efforts were made to engage with the most relevant and recent literature, including studies from the previous three years, this review reflects the current state of the field and highlights the need for further investigations that clearly delineate the unique roles and value of osteopathic care in sustainable healthcare systems.

In this context, the authors propose a research roadmap, aiming to stimulate future studies that support the reconceptualization and recontextualization of osteopathic principles within contemporary scientific, public health, and healthcare system frameworks. Finally, we acknowledge that one strength of our manuscript is the presentation of an OC model that is both distinctive and compatible with inter- and multidisciplinary collaboration. This model aligns with the core principles outlined in our perspective article, emphasizing holistic, patient-centered, and evidence-informed care. It is supported by literature demonstrating high patient satisfaction among individuals with musculoskeletal disorders receiving OC in integrated hospital settings, where satisfaction is influenced by technical quality, continuity of treatment, and accessibility [129]. Moreover, healthcare professionals recognize OC as a complementary discipline that can enhance team collaboration and patient care, underscoring the importance of structured protocols, interdisciplinary training, and integration within hospital-based practice [130]. Such collaborative approaches have the potential to improve patient outcomes across prevention, recovery, and rehabilitation programs, while also fostering effective communication, coordinated care, and overall service quality. Additionally, the narrative selection is not exhaustive and was guided by thematic saturation, conceptual relevance, and the expertise of the research team. Reflexivity was maintained throughout the review process, with continuous discussion among authors to critically evaluate the significance of included studies and ensure that interpretations are transparent, justified, and grounded in evidence. Although contemporary conceptual models, including the FEP, enactivism, complexity science, and systems medicine, offer valuable frameworks for understanding osteopathic care, their empirical validation remains limited. This highlights the need for further research to substantiate these theoretical approaches and to guide their application in evidence-informed, interdisciplinary care. This approach, while not eliminating all bias, strengthens the methodological and conceptual rigor of the Perspective.

## 6. Future Directions

The reconceptualization of osteopathic principles proposed in this paper requires empirical validation and a structured, ongoing dialogue within the profession. If osteopathy is to position itself coherently within prevention-oriented and sustainability-focused healthcare systems, future research must move beyond theoretical discussion toward operational validation along two complementary trajectories. The first trajectory emphasizes achieving international consensus on the interpretation and operationalization of osteopathic principles. This process should progress from conceptual exercises to collaborative inquiry, establishing shared definitions and clinically applicable frameworks to reduce interpretive variability. As outlined in the proposed research agenda (Appendix A, Table A1), this trajectory involves a sequential methodology, ranging from national surveys and qualitative focus groups to formal Delphi studies, aimed at constructing a shared interpretive framework that accommodates contextual diversity while preserving conceptual coherence. Such efforts are essential for reinforcing osteopathic identity and facilitating its integration within global healthcare education and practice. The second trajectory examines the systemic role of osteopathic care (OC) within local and interprofessional healthcare systems. Beyond consensus-building, research must address the practical implications of osteopathic practice as a “frugal,” low-technology, non-pharmacological modality. This approach is particularly suited to sustainability-oriented models, where clinical encounters function as micro-contexts for enhancing patient agency and promoting salutogenic behaviors, such as restorative sleep and lifestyle adaptation, without contributing to over-medicalization [3].

To support this, a dedicated research agenda is required to investigate the impact of osteopathic interventions on healthcare utilization patterns, quaternary prevention strategies, and their role within multidisciplinary teams (Appendix A, Table A2). Osteopathy, together with other therapeutic touch practices, is a key component of Touch Medicine, an emerging interdisciplinary field [131]. Evidence indicates that these interventions can produce antidepressant, anxiolytic, and analgesic effects through interoceptive, endocrinological, stress-related, and psychological mechanisms, offering a theoretical rationale for interpreting osteopathic effects in terms of predictive coding, the enactive model, the FEP [30,32], systems theory, complexity science [31,46], and salutogenesis [3], although direct clinical evidence remains limited. Future research should rigorously test these hypotheses in controlled studies, evaluate effectiveness on a larger scale, and translate findings into clinical guidelines to clarify the role of osteopathy across multiple healthcare specialties, including pediatrics, geriatrics, psychiatry, psychosomatic medicine, musculoskeletal and human movement medicine, and occupational medicine [131]. Collectively, these trajectories outline a roadmap for aligning osteopathic identity with contemporary healthcare priorities, fostering a model of care that is ecologically attuned, evidence-informed, and deeply integrated into public health frameworks.

## 7. Conclusions

This conceptual perspective has proposed a reconceptualization of osteopathic principles within contemporary scientific, public health, and healthcare system frameworks. Rather than marking a rupture with tradition, this process reflects osteopathy’s historical capacity for adaptation to evolving knowledge, societal expectations, and regulatory environments. In this sense, untangling the “Gordian knot” of osteopathic identity does not require abandoning foundational principles, but clarifying their meaning within present-day clinical, epistemological, and policy contexts. By situating osteopathy within prevention-oriented and territorial healthcare models, the paper has highlighted its potential relevance as a person-centered, low-technology, and non-pharmacological professional resource. Within such frameworks, osteopathy may contribute to reducing over-medicalization, supporting adaptive capacity, and aligning with sustainability-driven health policy priorities, while remaining complementary to, rather than competitive with, other healthcare professions. The Italian regulatory context illustrates how national recognition and prevention strategies can create structured opportunities for integrating osteopathy into public health-oriented systems. However, integration requires more than legislative acknowledgment. If osteopathy is to contribute meaningfully to prevention and territorial care, its education must be embedded within rigorous, publicly accountable academic structures aligned with national and international health priorities. Ultimately, reconceptualizing osteopathic principles offers an opportunity to strengthen professional coherence, enhance interprofessional credibility, and foster evidence-informed integration within sustainable healthcare systems. Future research should therefore prioritize structured consensus-building and staged empirical investigations capable of informing both policy development and clinical practice within prevention-oriented care pathways.

## Figures and Tables

**Table 1:** Summary of selected articles by thematic area.

Thematic Area	Electronic Databases and Digital Archives	Included Articles	Numbers of Articles
**Principles: History and Evolution** [2,12,13,14,15,16,17,18,19,20,21,22]	**Ostmed** and the **Internet Archive**	12, 13, 15, 16	12
	Pubmed	2, 14, 17, 18, 20, 22	
	Scopus, Google Scholar	19, 21	
**Reconceptualizing Osteopathic Principles for Applied Interdisciplinary Care** [23,24,25,26,27,28,29,30,31,32]	Pubmed	23–32	10
**Total**		2, 12–22, 23–32	22

**Table 2:** Principles: History and Evolution.

Theme	Historical Evidence	Key Insights/Clinical Interpretation
**Early definition of osteopathic principles**	Andrew Taylor Still’s *Our Platform* (1902) articulated nine statements to support health and combat disease [12]	Established foundational framework; need for conceptual clarity recognized early in the profession
**Divergence among practitioners**	Barbers and other early graduates interpreted principles differently [14]	Demonstrates long-standing debates on meaning and application of osteopathic principles
**Professional codification**	Codification of principles in 1922 [15] and condensation in 1953 [16]; reaffirmed in 2002 and 2005 emphasizing person-centered care [17,18]	Codification supported professional identity and consistent application of OMT
**Core tenets today**	Body as unified entity, self-regulation/self-healing, structure–function interrelation, treatment grounded in these principles [19,20]	Provides guiding framework; interpretations vary between traditional and contemporary scientific paradigms
**Adaptation to medical advances**	Although some authors argue that osteopathic professionals still hold reservations regarding certain practices, such as immunization, based on osteopathic tradition and the founder’s statements [21], historical evidence indicates that as early as 1910, while A.T. Still was still president, the American School of Osteopathy had already accepted the use of pharmaceuticals, anesthetics, antiseptics, and vaccines [22].	Illustrates osteopathy’s adaptability and integration with evolving medical knowledge

**Table 3:** Reconceptualizing Osteopathic Principles for Applied Interdisciplinary Care.

Theme	Historical/Conceptual Evidence	Key Insights/Clinical Interpretation
**Principles aligned with broader medical/philosophical traditions**	Similarities with indigenous healing, salutogenic models [23,24]	Osteopathy articulates effective clinical applications of shared concepts
**Self-regulation/self-healing**	Concept of *vis medicatrix naturae*, reinterpreted via psychoneuroendocrinoimmunology, personalized medicine, systems medicine [25,26]	Supports systemic adaptive capacity; therapeutic focus beyond reductionist mechanical interventions
**Structure–function relationship**	Historical roots in Aristotelian anatomy, Darwinian evolution; dynamic, adaptive biological systems [27,28,29,30]	Structural–functional integration underpins adaptive organism responses
**Therapeutic application**	OMT addresses movement patterns and somatic dysfunctions, supporting regulation [31,32]	OMT as strategy for systemic adaptation, rather than purely mechanical technique
**Biopsychosocial-existential unity**	Fourth osteopathic principle grounded in first three principles [31,32]	Emphasizes musculoskeletal system as primary interface for adaptive interaction, shaping perception, agency, and outcomes

**Table 4:** Glossary of Osteopathic Principles Through the Lens of Contemporary Conceptual Models.

Term	Academic Definition	Conceptual Framework
**Body (Soma)**	Biological and kinesthetic interface of human experience; a dynamic, autopoietic system manifesting individual adaptability through tissue representations and movement variability.	Enactivism, Kinesthesis [23,24,30,32]
**Mind**	Cognitive process of sense-making and verbal-nonverbal narrative integration; an emergent property of the interaction between bodily representations and environmental context.	Science of Complexity (i.e., Cynefin Framework) [23]
**Existence (Spirit)**	The term existence is used here to operationalize the principle historically referred to as spirit, reflecting the existential domain that encompasses the pursuit of meaning, purpose, and interconnectedness, and serving as a salutogenic pivot for biological and psychological resilience.	Salutogenesis; BPS Model [23,24]
**Self-Regulation**	Systemic capacity for allostasis and homeostatic maintenance mediated by neuro-endocrine-immune pathways to preserve health and minimize physiological entropy.	Systems Medicine; Psychoneuroendocrinoimmunology [26,31]
**Structure–Function**	Reciprocal, dissipative interaction where morphological form and physiological activity co-evolve to minimize free energy and reduce environmental unpredictability.	Free-Energy Principle [29,30,32]

## Data Availability

Not applicable.

## Abbreviations

The following abbreviations are used in this manuscript:

BPS	Biopsychosocial model
FEP	Free-Energy Principle
OC	Osteopathic care
OMT	Osteopathic Manipulative Treatment
PNEI	Psychoneuroendocrinoimmunology
SD	Somatic Dysfunction
SDGs	Sustainable Development Goals

## Appendix A

The reconceptualization of osteopathic principles proposed in this paper requires empirical consolidation through a structured research agenda (Table A1). This process should ideally move through sequential stages of consensus-building, starting with national and international surveys to assess agreement within the professional community, followed by qualitative inquiries to explore the practical barriers to integration. The use of Delphi methods and consensus workshops would be instrumental in finalizing a shared framework that preserves conceptual coherence while accommodating contextual diversity. Furthermore, research must address the systemic implications of osteopathic practice within prevention-oriented and territorial healthcare systems (Table A2). Future studies should investigate the contribution of osteopathic care to quaternary prevention and its impact on healthcare utilization patterns. By exploring the role of osteopathy in enhancing health literacy and supporting sustainable, interprofessional care pathways, the profession can align its identity with contemporary public health priorities. Such efforts would transition osteopathy from a purely clinical modality to a proactive resource for healthcare system resilience.

**Table A1:** Research Agenda for Achieving Consensus on Osteopathic Principles.

Aim	Type of Study	Description
Step 1: National Survey on Osteopathic Principles	Survey Study (Cross-sectional, quantitative)	Conduct a nationwide survey targeting the osteopathic community (practitioners, educators, and researchers) to assess their agreement on the emerging construct of osteopathic principles from the perspective paper. This survey will measure the level of consensus, identify discrepancies, and gather feedback on the reconceptualization process.
Step 2: Qualitative Interviews and Focus Groups	Qualitative Interviews and Focus Groups (Qualitative, explorative)	Follow up the survey with qualitative interviews and focus groups among a subset of survey participants. This will provide deeper insights into the reasons behind their agreement or disagreement with the proposed principles, as well as highlight any challenges or barriers to their integration into practice.
Step 3: Delphi Study	Delphi Study (Expert consensus, iterative)	Use the Delphi method to further refine the proposed osteopathic principles. This iterative process will engage a panel of experts to reach a consensus on the most significant and actionable principles for contemporary osteopathic practice, considering both theoretical and practical aspects.
Step 4: Comparative International Analysis	Comparative Study (Cross-national analysis)	Conduct a comparative analysis of the findings from the survey and Delphi study with similar studies in other countries or osteopathic communities to assess whether the emergent principles align with international practices or if cultural and contextual differences influence the consensus.
Step 5: Consensus Workshop and Framework Finalization	Consensus Workshop (Collaborative, participatory)	Organize a consensus workshop, bringing together key stakeholders from the osteopathic community, including educators, practitioners, and researchers, to finalize the shared framework of osteopathic principles. This workshop will involve discussions, revisions, and validation of the emerging framework based on the findings from previous steps.
Step 6: Publication and Dissemination	Consensus Study	Publish the final consensus framework in peer-reviewed journals and present it at national and international osteopathic conferences. Dissemination efforts will focus on ensuring that the new framework is accessible and applicable to osteopathic practitioners, researchers, and educators globally.
Step 7: Dissemination through Continuing Professional Development (CPD) Courses	Continuing Professional Development (CPD) Courses	Develop and offer CPD courses to disseminate the content of the consensus study within the osteopathic community. These courses will help integrate the agreed-upon principles into clinical practice and educational settings, fostering widespread adoption and application of the consensus framework.

**Table A2:** Research Agenda on Sustainability, Prevention, and Territorial Healthcare Systems: Focusing on Interprofessional Roles within the Osteopathic Profession.

Aim	Type of Study	Description
Sustainability of Healthcare Systems	Scoping Review, Systematic Review	Analyze existing literature on the sustainability challenges faced by contemporary healthcare systems, with a focus on aging populations, chronic diseases, and multi-morbidity.
Sustainability of Prevention Models	Observational, Cohort study and Prospective Longitudinal Study.	Investigate the long-term impact of prevention strategies on healthcare system productivity, focusing on cost reduction and system efficiency.
Territorial Healthcare System Resilience	Case Study	Examine the implementation of territorial healthcare systems in different regions, assessing how they contribute to sustainable healthcare delivery and the prevention of healthcare system overload.
Enhancing Health Literacy and Self-Management	Intervention Study (RCT, Observational Cohort and Case–Control Studies)	Test and evaluate different strategies for improving health literacy and self-management among citizens, focusing on their ability to reduce healthcare utilization and manage chronic conditions effectively.
Quaternary Prevention and Healthcare Over-medicalization	Economic Evaluation Study, Qualitative Study	Investigate the role of quaternary prevention in reducing over-medicalization and its impact on both individual health outcomes and the sustainability of healthcare systems.
Osteopathy’s Role in Territorial Healthcare	Cross-sectional Survey	Survey the role of osteopathy in territorial healthcare, particularly in community-based prevention and chronic disease management, and evaluate its effectiveness in reducing healthcare demand.
Interprofessional Collaboration in Prevention	Collaborative Practice Case Study	Explore the benefits of interprofessional collaboration, with osteopathy as a complementary resource, within prevention-oriented models of care. Assess how interprofessional practices can improve patient outcomes and reduce unnecessary medical interventions.
Workforce Development in Prevention-Oriented Professions	Policy Analysis	Assess the current state of education and workforce training for prevention-oriented healthcare professions, focusing on the integration of such roles into territorial healthcare systems. Examine policy frameworks supporting public investment in these professions.
Public Health Investment in Education for Prevention Roles	Economic Evaluation Study	Conduct an economic evaluation of public funding for education in prevention-oriented professions, evaluating the return on investment in terms of healthcare system resilience and long-term sustainability.
Osteopathy Education for Territorial Healthcare	Longitudinal Study	Explore the impact of osteopathy education, focused on prevention, within university-based systems. Assess how well these programs align with the long-term needs of territorial healthcare systems and public health investment goals.
Integration of Osteopathy in National Health Plans	Policy Review	Review national health policies, such as Italy’s National Prevention Plan, to evaluate how osteopathy is integrated and its contribution to sustainability, prevention, and healthcare system resilience.
